# Encephalic *Schistosoma japonicum* resembles brainstem neoplasm: Case report and literature review

**DOI:** 10.3389/fneur.2022.990998

**Published:** 2022-09-15

**Authors:** Kang Wu, Hong Yu Zhao, Kai Shu, Ting Lei, Liang Zeng

**Affiliations:** Department of Neurosurgery, Tongji Hospital, Tongji Medical College, Huazhong University of Science and Technology, Wuhan, China

**Keywords:** neuroschistosomiasis, blood flukes, bilharzia, pseudotumor, granuloma, brainstem

## Abstract

Encephalic schistosomiasis is a rare and severe parasitic disease which manifests as granuloma formation around ectopic eggs that migrate to the brain. We present a rare case of a pseudotumoral form of *Schistosoma japonicum* in the brainstem that was initially misidentified as a malignant tumor. The patient presented with intermittent headaches, diplopia, and left limb weakness. Neurological examination revealed hypoesthesia of the left lower limb, limitation of right eye abduction, and decreased muscle strength of the left upper limb. The cerebrospinal fluid tested positive for antibodies against *S. japonicum*. After standard treatment for schistosomiasis, the patient achieved complete remission. This case highlights that encephalic schistosomiasis can occur in the brainstem and resemble a neoplasm on magnetic resonance imaging. Once diagnosed, however, complete remission is achievable by non-invasive medical treatment.

## Introduction

Schistosomiasis is a chronic parasitic infection that affects approximately 240 million people worldwide. The main forms of human schistosomiasis are caused by three commonly known species: *Schistosoma haematobium* (endemic in Africa and the Eastern Mediterranean), *Schistosoma mansoni* (endemic in Africa, the Middle East, and South America), and *Schistosoma japonicum* (endemic in East Asia) (1). *S. japonicum* usually causes encephalic disease, whereas the other two species usually cause myelopathy (2, 3). Schistosomiasis is estimated to have existed in China for >2,100 years (4). Despite great efforts to prevent and control schistosomiasis, approximately 120,000 infections were reported in China in 2014 (5). Cerebral involvement in schistosomiasis is relatively rarer than hepatointestinal and urinary tract involvement. However, studies have suggested that the disease may be largely underestimated (2). Cerebral involvement mainly occurs in the cerebellum and the frontal, temporal, and parietal regions of the cortex (2, 6), and granulomas caused by *Schistosoma* species in the brainstem are extremely rare. To our knowledge, only two brainstem *S. mansoni* cases and one presumptive diagnosis of *S. japonicum* have been reported to date (Table 1) (7–9). This report reviews a rare case of a brainstem *S. japonicum* in a patient who came from the Honghu Lake region of China, one of the most epidemic areas for *S. japonicum* in central China. The Encephalic *S. japonicum* was initially suspected to be a brainstem neoplasm, highlighting the importance of understanding its uncommon locations and presentations.

**Table 1:** Published cases of schistosomiasis involving the brainstem.

**Author**	**Sex**	**Age (years)**	**Site of contamination**	**Location of lesions**	**Species of schistosome**	**Diagnosis**
Devine et al.	M	43	Rwanda	Pons, Midbrain	*Schistosoma mansoni*	Serology + rectal mucosa histology
Rommel et al.	F	69	Uganda	Medulla oblongata	*Schistosoma mansoni*	Serology + rectal mucosa histology
Liu et al.	M	12	China	Midbrain	*Schistosoma japonicum*	Serology + follow-up MRI

M, male; F, female; MRI, magnetic resonance imaging.

## Case report

**Figure 1:**
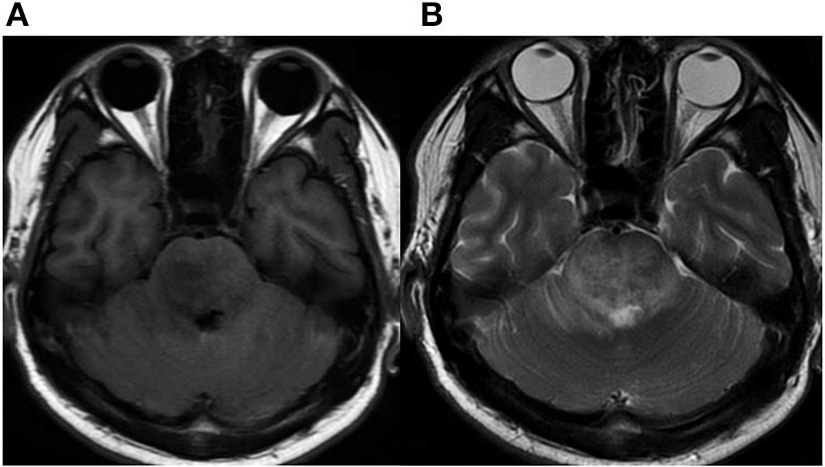
Initial MRI. **(A)** Axial MRI without contrast showing a hypointense signal on T1-weighted images in the right brainstem. **(B)** Intermediate signal on T2-weighted image surrounded by hyperintense signal of brain edema.

**Figure 2:**
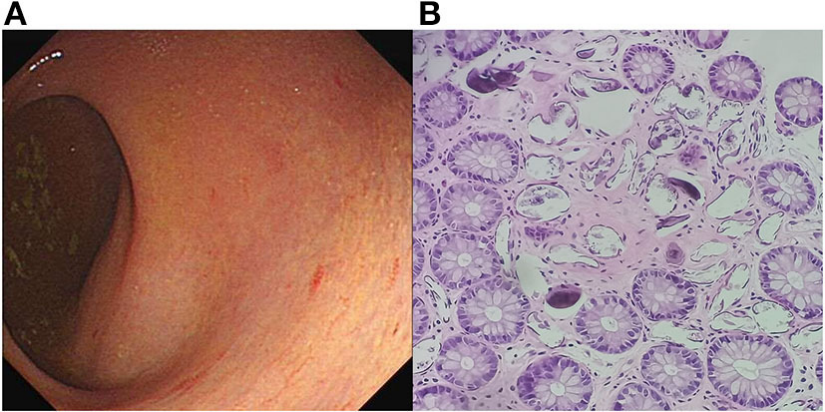
Colonoscopy imaging. **(A)** Yellow plaque formation under the enteric mucosa with an unclear blood vessel network, indicating a specific inflammatory response to *Schistosoma* eggs. **(B)** Photomicrograph of histologic section showing calcified *Schistosoma japonicum* eggs in the lamina propria of the rectum (hematoxylin–eosin staining, objective ×200).

**Figure 3:**
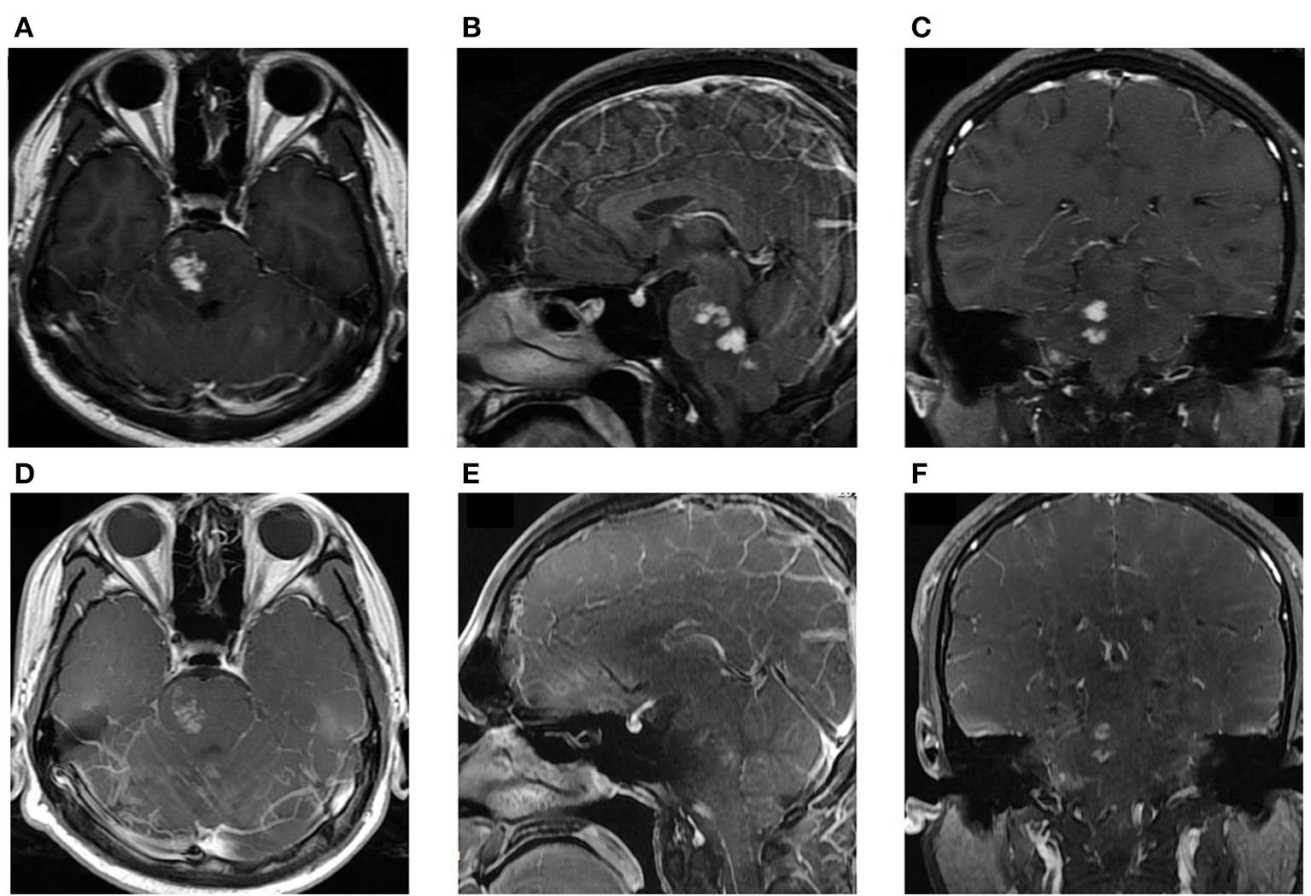
**(A–C)** MRI at admission: Axial, sagittal, and coronal T1-weighted, enhanced images showing significant, patchy, marked-enhancement lesions in the right brainstem after intravenous gadolinium administration. **(D–F)** Post-treatment MRI at the 4-month follow-up: T1-weighted enhanced images showing regressive lesions in the brainstem.

Written informed consent was obtained from the patient, and ethical approval was granted by the Institutional Review Board of the Affiliated Tongji Hospital of Huazhong Science and Technology University. A 25-year-old man was admitted to the emergency department with a 2-month history of intermittent headaches, diplopia, and left lower limb weakness. Cerebral magnetic resonance imaging (MRI) had been performed at the local hospital prior to presentation. T1-weighted without contrast images revealed a hypointense mass lesion in the right brainstem (Figure 1A). T2-weighted images showed an intermediate signal with irregular and poorly defined borders, surrounded by the hyperintense signal of edema (Figure 1B). These findings were suggestive of a slowly expanding malignancy, with a differential diagnosis of low-grade glioma. Therefore, the patient was transferred to a tertiary medical center. After he was admitted, neurological examination revealed hypoesthesia of the left lower limb and limitation of right eye abduction. The muscle strength of the limb was decreased to 4/5. The remaining physical and neurological examination results were unremarkable. Blood analysis demonstrated a normal leukocyte count of 6.52 × 10^9^/L with an elevated eosinophil count. His high-sensitivity C-reactive protein level was 8.0 mg/L (reference value: <3 mg/L). A blood sample enzyme-linked immunosorbent assay for parasite antibody IgG was positive for *S. japonicum* and negative for fascioliasis, toxocariasis, trichinellosis, cysticercosis, echinococcosis, and sparganosis. The cerebrospinal fluid (CSF) showed lymphocytic pleocytosis with 43 cells/μL. an elevated protein level of 827 mg/L, and IgG positivity for *S. japonicum*. An electronic colonoscopy showed a specific inflammatory response to the Schistosoma eggs (Figure 2A). The following mucosal biopsy confirmed the presence of *S. japonicum* eggs in the lamina propria of the rectum (Figure 2B). Gadolinium-enhanced T1-weighted sequencing showed areas of nodular enhancement in the brainstem, particularly on the right side. A non-specific tumor-like lesion that was surrounded by edema and associated with mass effect and heterogeneous contrast enhancement was revealed (Figures 3A–C). Although frame-based and frameless stereotactic biopsies have been widely used in other parts of the brain, considering the high probability of injury to nuclei and fibers in the brainstem area, surgical biopsy is not the first choice. The patient was treated with praziquantel (60 mg/kg) divided into two oral doses per day; the dose was adjusted to 40 mg/kg 1 day later. He also received prednisolone 30 mg daily for 1 week, which was tapered by 5 mg weekly. After 12 days, his neurological symptoms had improved significantly. He was discharged well 2 weeks later and continued tapering his steroid therapy at home. At the 4-month follow-up, the patient's symptoms had completely resolved, and a repeat cerebral MRI showed dramatic regression of the contrast enhancement in the brainstem (Figures 3D–F).

## Discussion

According to current evidence, neuroschistosomiasis is caused by the deposition of *Schistosoma* eggs in the central nervous system (6). *Schistosoma japonicum* eggs are small and round; therefore, they can travel through the blood–brain barrier and reach the brain. However, *S. mansoni* and *S. haematobium* eggs, which are larger and bear protruding spines, are prone to deposition in the spinal cord (10). Therefore, *S. japonicum* causes more encephalic disease than the other two species. It is believed that there are two possible routes for eggs to reach the brain: the eggs can be carried to the central nervous system through the arterial system or *via* retrograde venous flow through the valveless Batson's plexus, or the eggs can be locally deposited by the anomalous migration of adult worms (11–13). The first route may result in a scattered distribution of eggs in the brain, which has been supported in autopsy cases (14). The second route was confirmed by the finding of adult worms in meninges surrounded by eggs (15). Based on the anatomy and diameter of the vessels around the brainstem, we speculated that *S. japonicum* eggs may reach the brainstem by retrograde venous flow, possibly *via* some petrous sinuses and veins.

Clinical manifestations are caused by the host's inflammatory response to antigens released by *S. japonicum* eggs. Granuloma formation may result from T cell-dependent reactions and brainstem tissue necrosis. Space-occupying lesions and peripheral edema can lead to focal signs and even life-threatening conditions. In patients with neuroschistosomiasis, the most common symptoms include headaches, limb weakness, sensory loss, and cranial nerve paralysis, depending on the site of the lesion. Although the signs observed in our patient matched the above description, his clinical manifestations were indistinguishable from those caused by other types of slow-growing brainstem lesions, making it difficult to diagnose neuroschistosomiasis.

MRI is considered to be a useful imaging modality for diagnosing cerebral granulomas. Typical findings comprise hypointense lesions on T1 without contrast and multiple grouped hyperintense lesions on T1-weighted images with punctate nodules on post-contrast enhancement, forming an “arborized” appearance. In our case, MRI showed a grouped, nodular, enhancement pattern, which is consistent with previous reports on neuroschistosomiasis (14, 15). This characteristic finding helped us make the diagnosis and may help avoid unnecessary invasive procedures in future cases.

Diagnosis of brainstem schistosomiasis poses a serious challenge. Serologic tests may not be a suitable diagnostic tool because they cannot distinguish past infection from active disease; this is especially important for patients with long-term residence in epidemic areas (16, 17). Ancillary diagnostic test results, such as eosinophilia in peripheral blood, chronic inflammation found using electronic colonoscopy, and CSF lymphocytic pleocytosis, helped with diagnosis but were still non-specific. The identification of granulomas using tissue biopsy is considered the gold standard for diagnosis (9). However, for brainstem lesions, the life-threatening risks and uncertain benefits should be carefully considered beforehand. Recent studies have indicated that molecular diagnostic assays with *S. mansoni*-specific real-time PCR may be a non-invasive gold-standard diagnostic tool for patients with suspected symptomatic schistosomiasis because of its superior sensitivity. Surgical treatment for patients with neuroschistosomiasis should be individualized because of the risk of additional damage to the involved nervous tissue (10). Although contemporary high-precision medical equipment, such as neuronavigation and electrophysiological monitoring are widely used, surgery or biopsy of brainstem schistosomiasis is still considered a high-risk procedure that may result in severe consequences (18).

Previous reports indicate that most cases of neuroschistosomiasis can be treated safely and effectively with praziquantel plus steroids (19), therefore, diagnostic anthelmintic therapy with informed consent was recommended to the patient. Praziquantel is an effective broad-spectrum vermifuge that can destroy adult worms and interrupt egg production. Steroids should be used in the early stages of neuroschistosomiasis to eliminate the inflammatory reaction and consequently relieve neurological symptoms (20, 21). The patient's pronounced and rapid improvement after therapy further confirmed this diagnosis.

## Conclusions

Brainstem schistosomiasis is an extremely rare disease that is difficult to diagnose. Clinical signs and plain MRI scans may be misleading, leading to an initial diagnosis of neoplasm. Heterogeneously enhancing lesions with surrounding edema on MRI make brainstem schistosomiasis a differential diagnosis. Other clinical, laboratory, and epidemiological findings further support the presumptive diagnosis of neuroschistosomiasis. The present case also indicated that surgery or biopsy of the brainstem should not be the first treatment of choice. Anti-schistosomal chemotherapy with praziquantel and steroids in the absence of a tissue-based diagnosis is feasible, safe, and effective.

## Data availability statement

The original contributions presented in the study are included in the article/supplementary material, further inquiries can be directed to the corresponding author.

## Author contributions

All authors listed have made a substantial, direct, and intellectual contribution to the work and approved it for publication.

## Funding

This work was supported by the Nature Science Foundation of HuBei Province (2021CFB378).

## Conflict of interest

The authors declare that the research was conducted in the absence of any commercial or financial relationships that could be construed as a potential conflict of interest.

## Publisher's note

All claims expressed in this article are solely those of the authors and do not necessarily represent those of their affiliated organizations, or those of the publisher, the editors and the reviewers. Any product that may be evaluated in this article, or claim that may be made by its manufacturer, is not guaranteed or endorsed by the publisher.
